# Active Volcanism Revealed from a Seismicity Conduit in the Long-resting Tatun Volcano Group of Northern Taiwan

**DOI:** 10.1038/s41598-020-63270-7

**Published:** 2020-04-09

**Authors:** H. C. Pu, C. H. Lin, Y. C. Lai, M. H. Shih, L. C. Chang, H. F. Lee, P. T. Lee, G. T. Hong, Y. H. Li, W. Y. Chang, C. H. Lo

**Affiliations:** 1 Seismological Center, Central Weather Bureau, Taipei, Taiwan; 2 Institute of Earth Sciences, Academia Sinica, Taipei, Taiwan; 3 National Center for Research on Earthquake Engineering, National Applied Research laboratories, Taipei, Taiwan; 4 Taiwan Volcano Observatory at Tatun, Taipei, Taiwan; 5 Central Geological Survey, Ministry of Economic Affairs, Taipei, Taiwan; 6 Industrial Technology Research Institute, Hsinchu, Taiwan; 7 College of Environmental Studies, National Dong Hwa University, Hualien, Taiwan; 8 Center for Interdisciplinary Research on Ecology and Sustainability, National Dong Hwa University, Hualien, Taiwan; 9 Department of Geosciences, National Taiwan University, Taipei, Taiwan

**Keywords:** Natural hazards, Seismology, Volcanology

## Abstract

Abundant earthquakes clustered within a particular zone often reflect an active geological feature, such as clustering seismicity along a fault zone and a huge number of volcanic-earthquakes around the erupting conduit. Herein we perform a double-difference tomographic inversion and relocate the seismicity at the long-resting Tatun volcano group (TVG) in northern Taiwan. A dramatic improvement of the earthquake location model surprisingly show that, from 2014 to 2017, two clustered seismic zones are identified in the TVG. One major group of events (>1000) persistently clustered within a ~500 m diameter vertical conduit with a ~2 km height. The clustering seismicity conduit is just located nearby Dayoukeng, one of the strongest fumaroles in the TVG, and is connected to a fracture zone characterized by low Vp/Vs in the shallow crust. The other group of events is clustered within a sphere-like zone beneath Mt. Chihsin around the depths between 0.5 km and 2 km. Both seismic zones are probably triggered by the significantly volcanic gases and fluids ascending from the deep magma reservoir. Combined with a variety of results from literature, the seismicity conduit near the strong fumarole is the evidence for an active volcano and also identifies a likely pathway for ascending magma if the TVG erupts again in the future. But possibility of developing different magma pathways at other clustered seismic zones such as beneath Mt. Chihsin may not be totally excluded.

## Introduction

The evaluation of whether or not a volcano will erupt often relies on the classification of it as active, dormant, or extinct; however, the exact definition of an active volcano may still be debatable. Two criteria are often employed to classify an active volcano: (1) the volcano erupted within the past ~10,000 years or (2) the identification of magma chambers beneath the volcano^1^. However, those definitions are not set in stone because some eruptions have surprisingly occurred in dormant or extinct volcanoes, which are long-resting volcanoes without evidence of any magma chambers beneath them. Thus, some other criteria may be involved in the evaluation of the possibility of eruptions in long-resting volcanoes, in particular.

**Figure 1:**
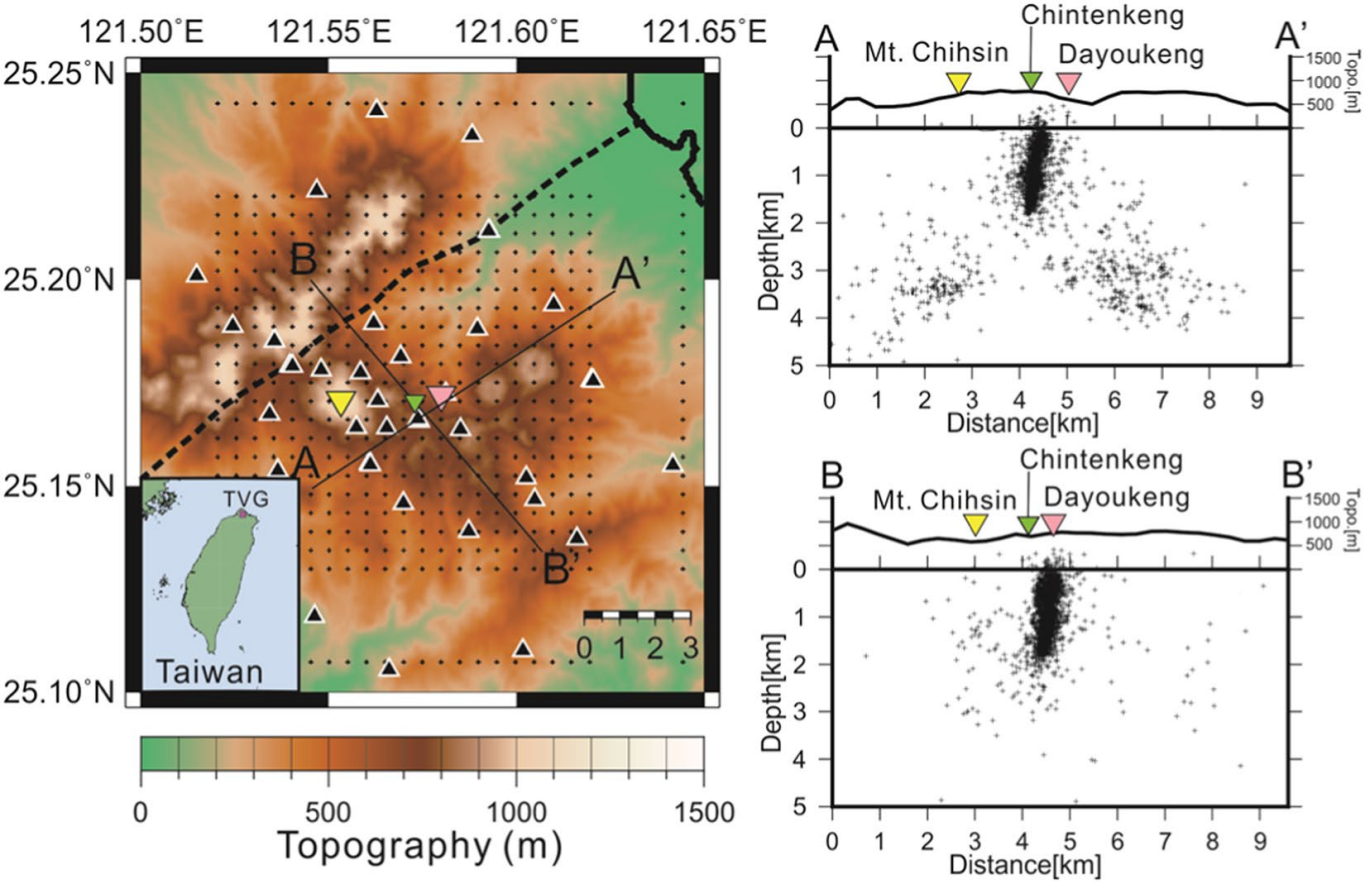
Locations of the Tatun volcano group (TVG) in the northern tip of Taiwan and two perpendicular depth-profiles across the Dayoukeng area to show the clustering seismicity (pluses) within a narrow vertical conduit at depths shallower than 2 km. The black, yellow, green and pink triangles in the left panel mark the locations of seismic stations, Mt. Chihsin, Chintenkeng and Dayoukeng fumarole in the TVG, respectively. The dashed line shows the surface trace of the Shanchiao fault. The relocated micro-earthquakes are plotted along the profiles within a width of 0.5 km.

The Tatun volcano group (hereinafter “TVG”) in the northern tip of Taiwan is a typical long-resting volcano because there was no eruption record in human history. From the volcanic hazard point of view, it is important to know whether or not the TVG is active because it is located near the Taipei metropolis, with more than 6 million residents living in both Taipei City and New Taipei City in northern Taiwan (Fig. 1). The distance between Mt. Chihsin (the highest peak of 1120 m in the TVG) and the Taipei 101 skyscraper building (a landmark in downtown of Taipei City^2^) is less than 15 km. In other words, the TVG is a typical “City on Volcano” case if it is active. Apart from the Taipei metropolis, it is worth highlighting the two nuclear power plants located around the northern boundary of the TVG. Thus, even a small volcanic eruption at the TVG might pose a major threat to the Taipei metropolis due to direct volcanic disasters, such as lava or pyroclastic flows and volcanic ashes, and also social and economic impacts in Taiwan. Therefore, detailed volcanic studies and monitoring works are two important tasks if the TVG is an active volcano group.

A lot of volcanic features such as fumaroles and hot-springs are still significant at the TVG, but it was considered to be dormant or even extinct for a long time. Early stage geological studies showed that the volcanic eruption primarily started around 0.8 Ma and eventually stopped around 0.1 Ma^3–5^. However, a variety of recent observations and analyses suggest that the TVG may be still active. First, Helium isotopes indicate that some mantle signatures have been detected from the volcanic gas and fluid at many fumarole sites^6^. Second, shallow pressure sources, including both inflation and deflation, have been detected from high-accurate leveling surveys across the TVG^7^. Third, clustered micro-earthquakes with some typical volcano-earthquakes, such as tornillos, mono-chromatic tremors, long-period events, and heartbeat earthquakes, have been observed in the shallow crust beneath the TVG^8–12^. Furthermore, a few small-scale phreatic eruptions have been detected by infrasonic sensors and seismic arrays^13,14^. Fourth, the dating results from volcanic ashes and Fe-rocks imply that the last eruption took place within several thousand years^15,16^. Finally, a deep magma reservoir has been detected through the seismic detection of both S-wave shadows and P-wave delays^2^.

All of the late observations and analyses mentioned above suggest that a future volcanic eruption in the TVG cannot be totally excluded; however, the detailed subsurface structures, such as shallow hydrothermal systems, volcanic conduits, and deep magma reservoirs, necessary to delineate the geometry and connections between volcanic plumbing systems are still unknown. Herein, we produce seismic tomographic images based on the P- and S-wave arrival time data recorded at 40 broadband seismic stations in the TVG area between 2014 and 2017. The 3-D velocity structures of P-waves (Vp), S-waves (Vs) and the ratios between both (Vp/Vs) at depths shallower than 5 km can successfully be inverted by the double-difference tomography^17^. These tomographic images allow us to improve the understanding of the volcanic plumbing system in the shallow crust beneath the TVG. Also the relocated seismicity interestingly shows a vertical conduit within a ~500 m diameter area near the Dayoukeng fumarole, one of the strongest degassing processes in the TVG, at a depth between ~0.2 to 2 km below the surface. Such a seismicity conduit is the major pathway for the transport of volcanic gases and fluids from deep reservoirs to the surface; it may be one of the most likely pathways for ascending magma for TVG eruptions in the future. But possibility of developing different magma pathways at other clustered seismicity zones such as beneath Mt. Chihsin might not be totally excluded. These results significantly help in the understanding of the TVG plumbing system and provide some critical information to mitigate future volcanic hazards in the Taipei metropolis.

## Results (High-Resolution Seismic Tomography)

We perform the double-difference seismic tomographic inversion (tomoDD) of the P- and S-wave arrivals to more than 2,000 micro-earthquakes recorded by 40 broadband seismic stations in the TVG in northern Taiwan between 2014 and 2017. The seismic array was installed and gradually upgraded by the Taiwan Volcano Observatory at Tatun (TVO), and the P- and S-wave arrivals have been routinely picked for locating earthquakes by using the traditional algorithm (Hypo71) based on a simplified layer velocity model since 2003^8,9^. The preliminary results of seismicity show that two groups of earthquakes are roughly clustered at the shallow crust just beneath Mt. Chihsin and the Dayoukeng fumarole^9^. Mt. Chihsin is the highest peak and probably the youngest volcano in the TVG^15^ and the Dayoukeng fumarole is one of the strongest degassing processes in the TVG [^12^; supplemental video].

**Figure 2:**
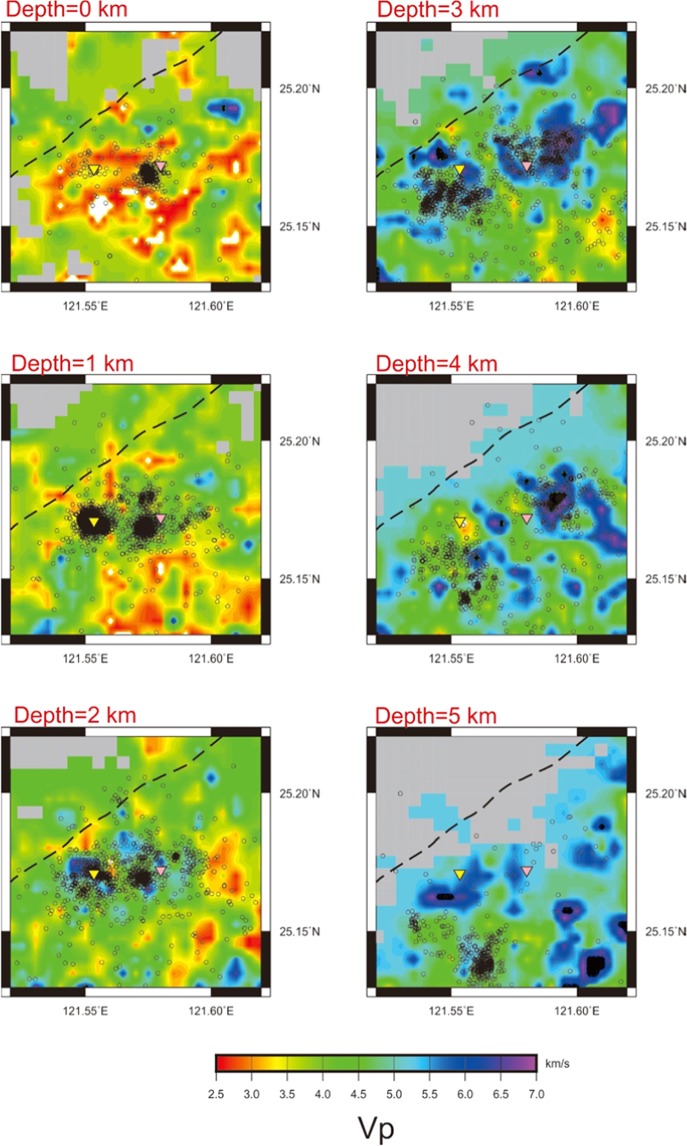
P-wave Velocity structures at six layers from 0 km to 5 km above the sea level. The relocated seismicity (small circles) is also plotted. The dashed line, yellow and red triangles, respectively, show the Shanchiao fault, Mt. Chihsin and Dayoukeng fumarole here and the following figures.

**Figure 3:**
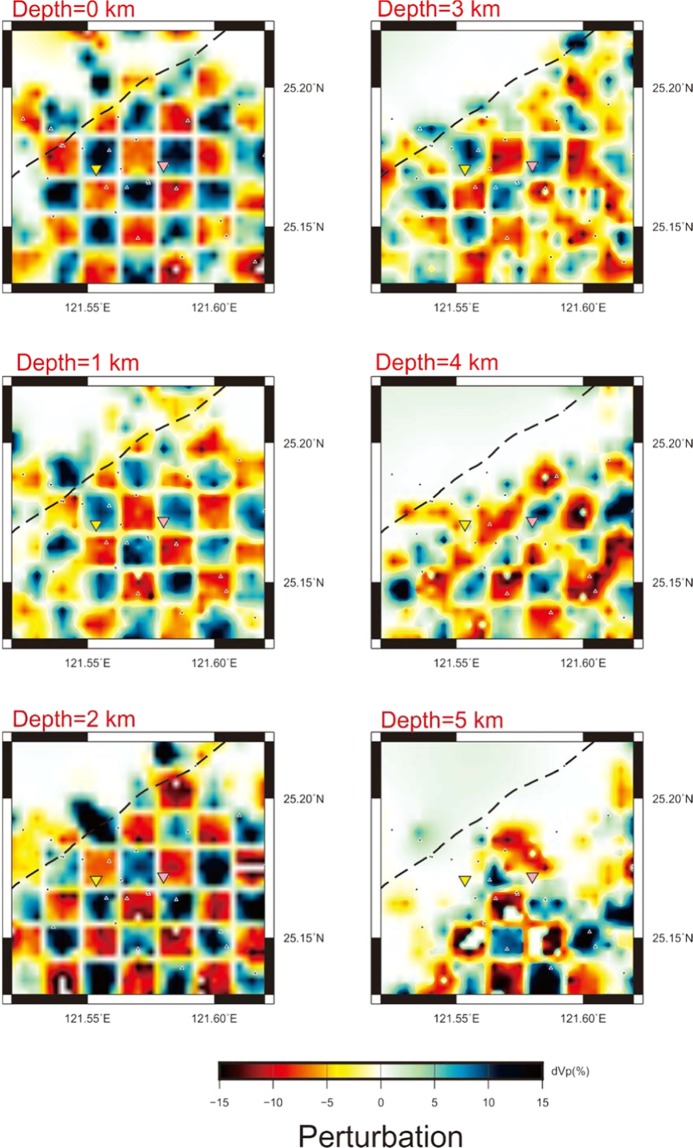
The P-wave perturbations in 6 layers inverted from the checkerboard velocity model.

The inverted P-wave and S-wave velocity models (Vp and Vs) at 6 different depths from 0 km (sea level) to 5.0 km are shown in Figs. 2 and S1, respectively. As expected, a strong heterogeneity of both Vp and Vs are observed at the shallow crust due to the variations of rock types (lithology), of fractures, and in temperature, and probably also the volcanic fluids or gases involved. In general, low Vp zones, shown in red, are broadly detected at the shallow depths (<2.0 km), while high Vp areas, shown in blue, are largely at deep depths (>3.0 km). For areas with Vp less than 3.0 km/s, they are likely reflected on fractural rocks filled with hydrothermal fluids or volcanic gases because a lot of hot-springs and fumaroles are observed in the Tatun volcanic area^18,19^. Based on the geology survey^20,21^, the andesitic rocks largely observed on the surface is overlay on the sedimentary rocks in the Tatun volcanic area. Thus, areas with Vp greater than 5.0 km/s are associated with andesitic rocks that intruded into sedimentary rocks at the previous volcanism. The general features from the tomographic inversion are reliable because major portions of the study area can be successfully inverted by checkerboard tests due to the high density of ray-paths from the abundant micro-earthquakes at the shallow depths (Figs. 3 and S2). However, both the structural heterogeneity and the inversion reliability gradually decrease with depth because of the limited ray-paths in the deeper layers.

**Figure 4:**
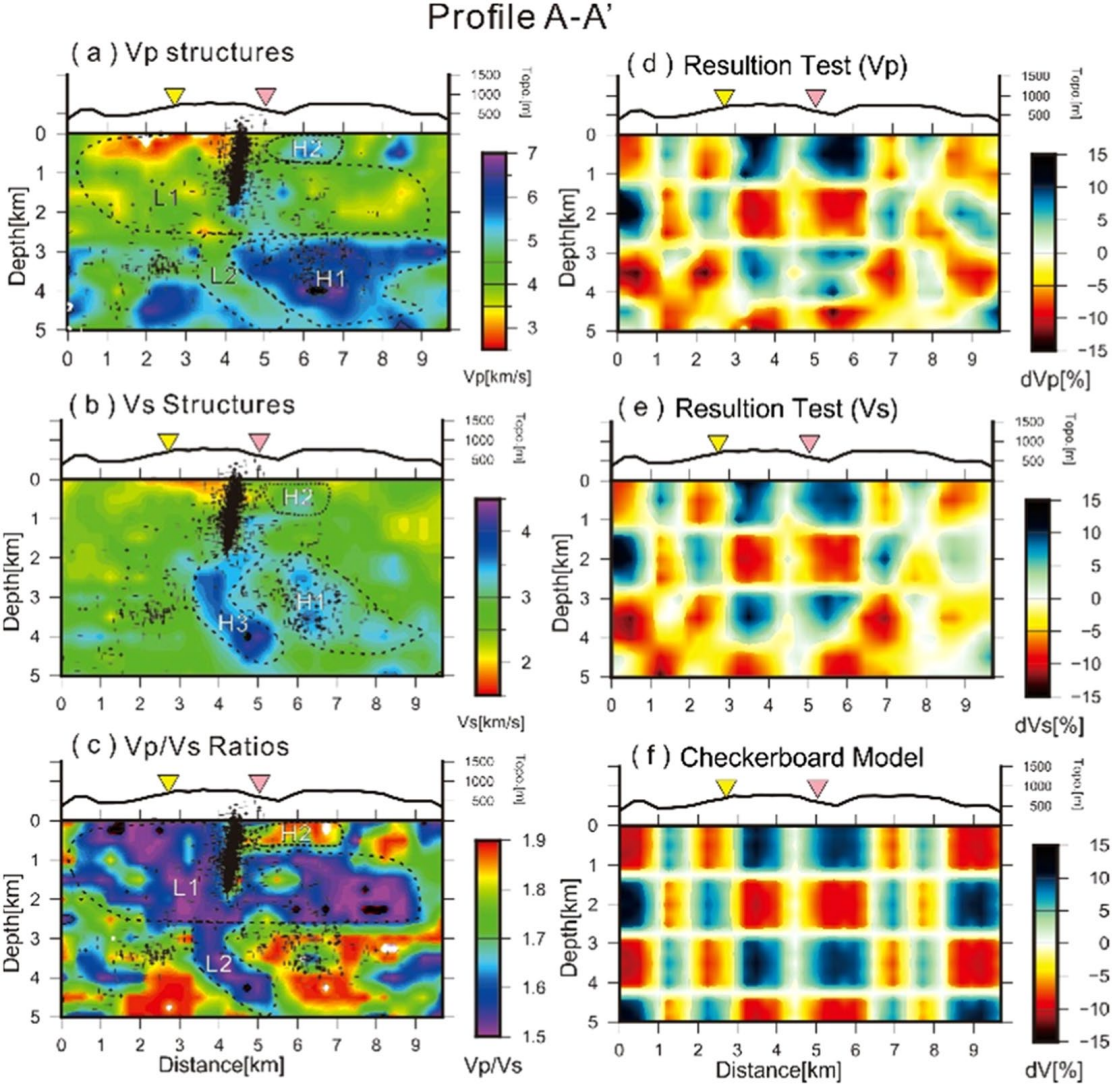
The inverted results of (**a**) Vp, (**b**) Vs, and (**c**) Vp/Vs ratio structures as well as the checkerboard tests of (**d**) Vp and (**e**) Vs based on (**f**) the assumed velocity model across the A-A’ profile shown in Fig. 1. Dashed lines delineate the velocity anomalies of low (L1 and L2) and high (H1, H2, and H3) zones.

**Figure 5:**
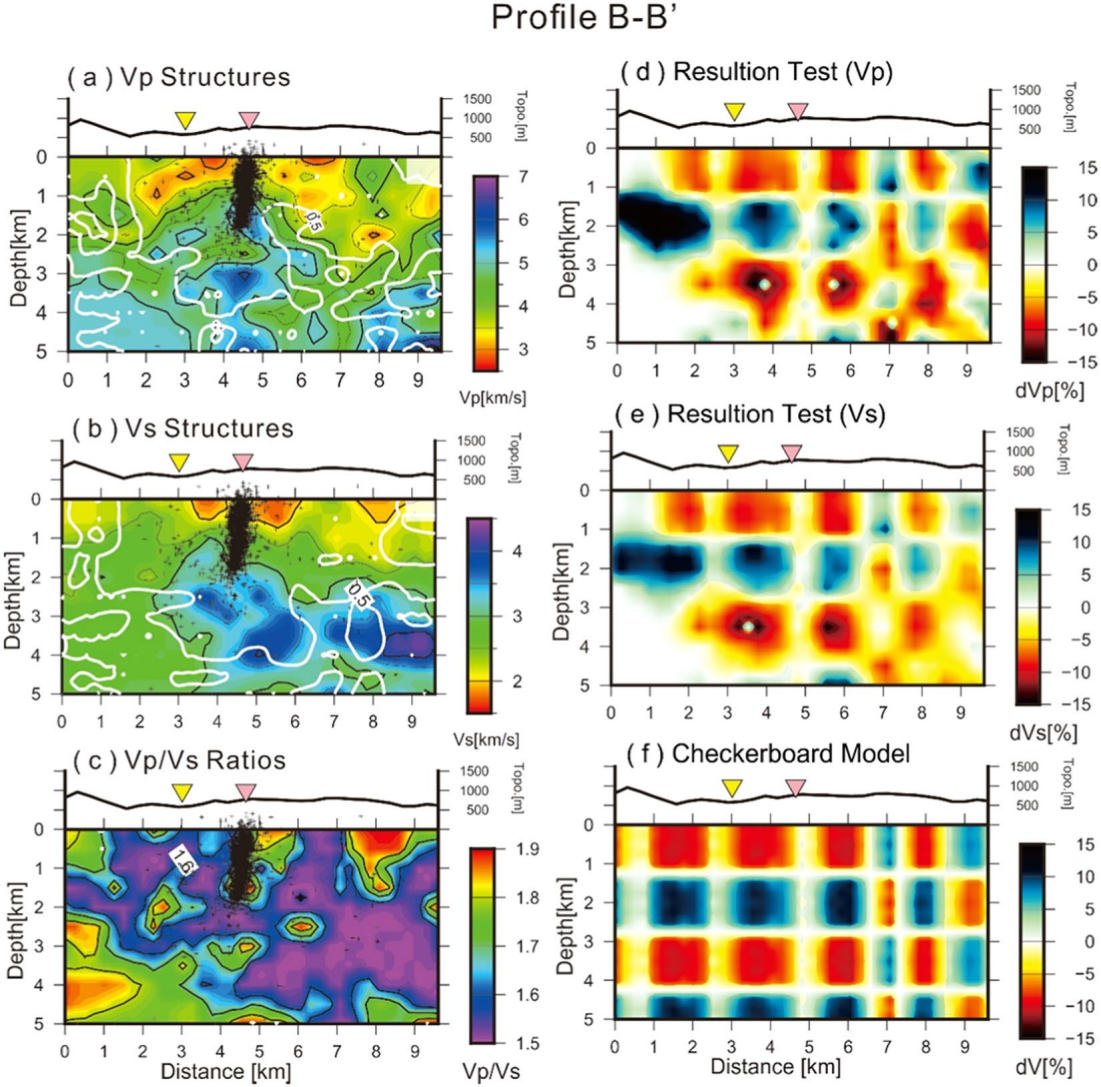
**(a**) Vp, (**b**) Vs and (**c**) Vp/Vs ratios as well as the inversed results of (**d**) Vp and (**e**) Vs based on (**f**) the assumed checkerboard model across the D-D’ profile shown at Fig. 4.

To discuss the detailed geological features beneath the TVG, a representative velocity profile (A-A’) across the Dayoukeng area is plotted in Fig. 4. Again, major portions of the velocity structures are successfully inverted according to the checkerboard test (Fig. 4d,e). The assumed checkerboard model with velocity variations of 10% in each layer (Fig. 4f) are basically obtained in most grids for both the Vp and Vs models after the tomographic inversion. These checkerboard tests suggest that major portions of the inverted velocity structures are reliable for the delineation of the subsurface structures along the A-A’ profile due to dense seismic ray-paths. Similar results across major portions of the study area are shown along Profile B-B’ (Fig. 5), which is almost perpendicular to Profile A-A’ (Fig. 1).

The seismic tomographic images across the A-A’ profile show two prominent zones (L1 and L2) where low Vp and Vp/Vs ratios are identified at different depths (Fig. 4a,c). The shallow one (L1) is a broad area from the surface down to a depth of ~2.5 km, and the deep one (L2) is a narrow zone dipping to the northeast just beneath the Dayoukeng area. The areas with both low Vp and low Vp/Vs ratio are probably associated with supercritical fluids^21,22^, because Vp decreases as pore pressure in the saturated rocks increases^23,24^ and Vp/Vs decreases if a gas phase is added to the saturated fluid^25,26^.

Furthermore, a broad area just below the vertical seismicity conduit at depths below ~ 2.5 km shows high Vp and Vs (H1 in Fig. 4a,b), which are interpreted as andesitic rocks that intruded into sedimentary rocks at the last volcanism because the andesite and sedimentary are two of major rocks near the surface^20,21^. A lot of hot springs and fumaroles in and around the Dayoukeng area imply the hydrothermal activity is strong at the shallow depths^18,19^. In other words, high fluids and fracturing might be expected in the Dayoukeng area. The presence of both high fluids and fracturing will reduce Vs more than Vp, resulting in a high Vp/Vs ratio. Such high Vp/Vs ratios with high fluids and fracturing phenomena are likely caused by the upwelling of magma fluids and the heat from the deep magma reservoir in the lower crust^2^. Another small area (H2) with high Vp, Vs, and Vp/Vs is shown at extremely shallow depths (0–800 m) beneath the Dayoukeng area, which can also interpreted as high fluids and fracture features within andesitic rocks.

**Figure 6:**
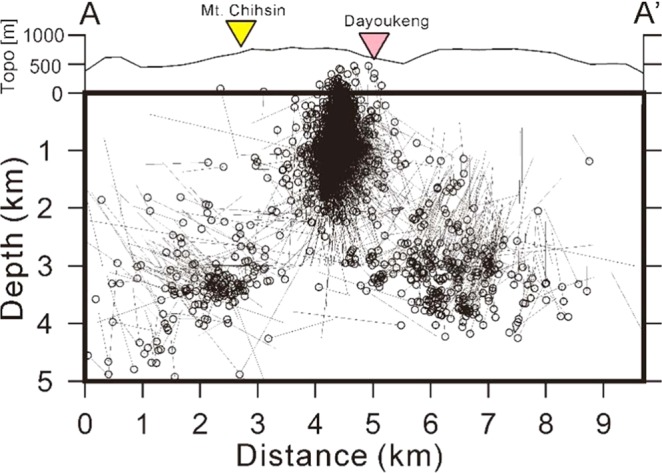
The hypocenter shift distances (dashed lines) before and after (circles) the relocation process across the A-A’ profile.

**Figure 7:**
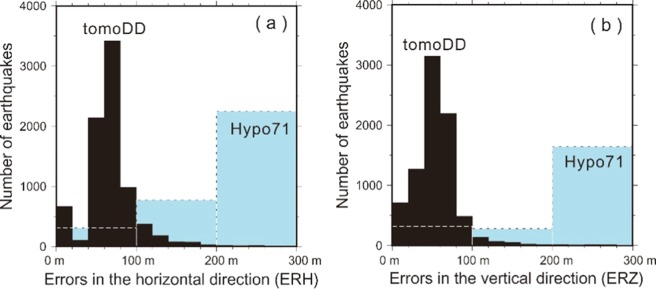
Statistical comparisons of hypocenter errors for both the (**a**) horizontal and (**b**) vertical directions before (Hypo71 in blue) and after (tomoDD in black) the relocation process.

The relocated seismicity by tomoDD around the Dayoukeng fumarole is surprisingly clustered at an extremely narrow vertical conduit, which is limited to a small area of approximately 0.5 km in diameter from a few of hundred meters below the surface down to ~2 km in depth (Fig. 1). In contrast, there are only very limited earthquakes (less than 10%) away from this seismicity conduit in and around the Dayoukeng area. Similarly, the seismicity beneath Mt. Chihsin is largely clustered within a sphere-like zone around the depths between 0.5 and 2.0 km (Fig. S3). In general, it is very surprising to see that the locations of many micro-earthquakes have been significantly improved through a careful relocation process based on the inverted 3-D velocity model (i.e., Fig. S4). The relocated seismicity beneath the Dayoukeng area can be generally divided into three major groups along the A-A’ profile (Fig. 6). For the seismicity northeast of the Dayoukeng area, the relocated earthquakes are generally shifting southwest and increase to a depth of about 1–2 km. On the other hand, the relocated earthquakes southwest of the Mt. Chihsin area are shifting in the northeast direction. It is more interesting to see that the relocated earthquakes between the Mt. Chihsin and Dayoukeng areas are clearly clustered into the narrow vertical conduit. In particular, some of the focal depths beneath the Dayoukeng area are moved to above the sea level. Obviously, such a dramatic improvement is associated with not only the significant change of the strong heterogeneity from the absolute arrivals of both P- and S-waves, but also the huge number of relative arrivals of both P- and S-waves for reducing systemic errors. Certainly, the relocated seismicity is more reliable because the estimated errors of the earthquake locations in both the horizontal (ERH) and vertical (ERZ) directions are significantly improved after the relocation process (Fig. 7). The statistical comparison before and after the relocation process shows that earthquakes with an error less than 100 meters dramatically increase from ~20% to ~90%.

## Discussion

**Figure 8:**
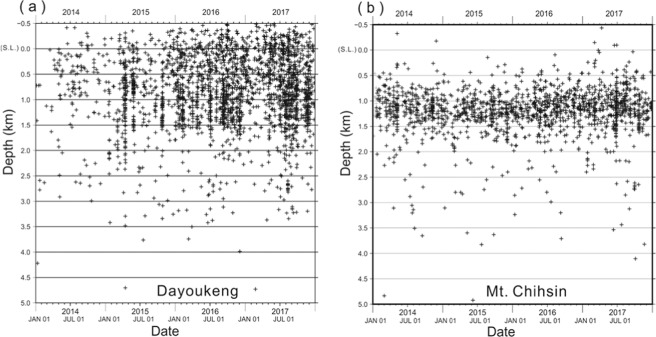
Plots of focal depths with time for showing seismicity is largely persistent through time at (**a**) the Dayoukeng area (121.565°E-121.58°E and 25.16°N −25.175°N) and (b) Mt. Chihsin (121.54°E-121.56°E and 25.16°N −25.18°N).

The highly clustered seismicity within an extremely narrow conduit suggests that the volcanic activity in the TVG is still ongoing. After the tomoDD relocation, the preliminary earthquakes diffused as a cloud of seismicity are dramatically clustered into an extremely narrow vertical conduit (Figs. 6 and S4). Although there are some possible small uncertainties for each individual earthquake location, the general pattern of seismicity clustering at a vertical conduit beneath the Dayoukeng area doesn’t change too much due to dense seismic ray-path covering at that area. Such a concentrated seismicity is probably caused by the stress changes within a shallow volcanic conduit in the TVG. This conduit might be composed of fractural rocks that are not only allowing volcanic fluids or gases easily went through, but also inducing micro-earthquakes. Since the seismicity was not mainly dominated by seismic swarms but persistent from 2014 to 2017 (Fig. 8), the seismicity conduit couldn’t been suddenly generated along a local fault due to stress transfer. Similar cases were reported at many active volcanoes, such as the Kilauea volcano in Hawaii^27,28^, Sakurajima volcano in Japan^29^, Mt. Vesuvius in Italy^30,31^, and Soufriere Hills in Montserrat^32^. Some of the concentrated seismicity can be directly associated with the magma movement, but others may reflect the background seismicity in the volcanic area. No matter the mechanism for triggering these volcano-earthquakes, the concentrated seismicity within a particular area often shows that a volcano is still active. Like in active faults, the concentrated seismicity is detected along their narrow fault zones (i.e.^33–36^). Therefore, the persistent and stable seismicity within a narrow conduit in the TVG indicates it is still active even though there have been no eruption records in human history.

Furthermore, it is interesting to see that the seismicity conduit (earthquakes clustered within a vertical conduit) is near the Dayoukeng area, which has one of the strongest degassing fumaroles on the surface (i.e., supplemental movie). A variety of interesting anomalies were observed in and around the Dayoukeng area from geochemical, geophysical, and seismic analyses in the past decade. Firstly, the strong degassing process at the Dayoukeng fumarole has created several large sulfur-towers (i.e., Fig. S5), which have undergone a repeating process of collapse and re-building from time to time. The tallest height of these sulfur-towers may be up to ~ 5 meter (Fig. in^19^). The detailed geochemical analyses of collected fluids and gases at all fumaroles in the TVG show that the Dayoukeng fumarole has a significant signature of sulfur compounds and hydrogen chloride as well as high helium isotope ratios (~6.8). These results strongly indicate that the degassing process is not associated with the hydrothermal but with magmatic activities^6,18,19^. Secondly, extremely high heat-flows have been detected in the Dayoukeng area^19^. The steam temperature measured at the Dayoukeng fumarole is around 105 °C at the surface and increases to 250 °C at a depth of ~200 meter within a borehole at Chintenkeng, which is less than 100 m from the seismicity conduit. Thirdly, the chemical signatures observed from the Helium isotope in the TVG show that the Dayoukeng area has the highest ^3^He/^4^He ratio (Yang *et al*., 1999), which implies the existence of magma chamber underneath this area. Fourth, some inflation sources have been detected by the crustal deformation as well as the seismic data. One inflation source at a depth of 0.7 km beneath the Dayoukeng area has been detected from repeated precise leveling surveys^7^. The inflation source within the pressurized hydrothermal layers may be created by the ascension of volcanic fluids from the deep magma reservoir. Similarly, another deeper inflation source has been identified around a depth of 2 km beneath the Dayoukeng area from 1016 earthquake focal mechanisms^37^. The stress magnitude ratios suggest this inflation is also associated with the local volcano-hydrothermal activity. Those shallow inflation sources might be caused by migrations of volcanic fluids and/or gases. Finally, a variety of interesting volcanic earthquakes and tremors have been observed in and around the Dayoukeng area, indicating strong degassing at shallow depths. For instance, a sequence of high-frequency spasmodic bursts within a duration of ~15 minutes was detected^9^. Also, heartbeat-like seismicity with a repeated period of ~18 minutes suggests a volcanic conduit may exist just beneath the Dayoukeng area^12^. Combining all the unusual features from the geochemical, geophysical, and seismic data with the significant degassing process on the surface, we may conclude that the Dayoukeng area is one of the major sites releasing the volcanic material and heat ascending from the deep magma reservoirs of the TVG.

The velocity structures obtained from the double-difference tomography indicate that the upwelling volcanic fluids and gases largely pass through some fracture zones beneath the Dayoukeng area. For instance, a northeast-dipping zone with low Vp/Vs (L2 in Fig. 4c) with depths between 3 km and 5 km may be one of the major pathways for ascending volcanic materials. Thus, major portions of the ascending material and heat may occasionally trigger clustered micro-earthquakes within a narrow vertical conduit at depths between 2 km and the surface. Meanwhile, other ascending materials and heat are migrating into broad hydrothermal zones (L1 in Fig. 4a,c) at depths shallower than ~2.5 km.

**Figure 9:**
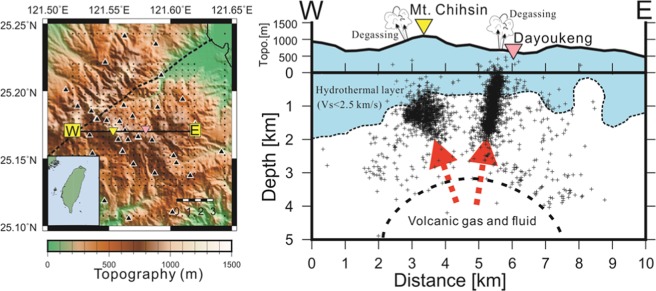
The map of the TVG (left) and a projection of seismicity across Dayoukeng and Mt. Chihsin along the W-E profile (right). The relocated micro-earthquakes are plotted along the profile within a width of 0.5 km. Schematic plots of the hydrothermal layer (Vs <2.5 km/s) at shallow depths as well as ascending volcanic gases and fluids are also added.

In addition to the seismicity clustered beneath the Dayoukeng area, the other group of micro-earthquakes is clearly identified beneath Mt. Chihsin (Fig. 9). Although the seismicity beneath Mt. Chihsin doesn’t show a conduit-like shape, most of micro-earthquakes are detected within a small sphere-like zone within a diameter of ~ 1 kilometer. Similar to the earthquakes at the Dayoukeng area, seismicity beneath Mt. Chihsin is steady and persistent (Fig. 8). Also their focal depths are roughly ranging from between 0.5 km and 2.0 km. Both groups of earthquakes at the Dayoukeng area and Mt. Chihsin might be taken place within a hydrothermal layer and likely induced by the volcanic gases and fluids ascending from the deep magma reservoir beneath the Tatun volcanic group.

In summary, the seismic conduit beneath the Dayoukeng area as well as the clustered seismicity beneath Mt. Chihsin may be considered as an important seismic evidence for an active volcano in the TVG. In particular, the clustered micro-earthquakes within a narrow ~500 m diameter conduit beneath the Dayoukeng area have been persistently triggered by the ascending volcanic gases or fluids, which were originally released from the deep magma reservoirs at the lower crust^2^ and passed through some fracture zones with low Vp/Vs values in the uppermost crust (Fig. 4). The magmatic signature was clearly shown by the significant sulfur compounds and hydrogen chlorides as well as high helium isotopes from the ascending gases and fluids near the Dayoukeng fumarole^6,19^. The pressurized hydrothermal layer at a shallow depth was revealed by an inflation source at a depth of 0.7 km^7^ and another deep volcano-hydrothermal inflation was identified at a depth of ~2 km^37^ beneath the Dayoukeng area. Also the spasmodic bursts^8^ and heartbeat seismicity^12^ have been observed beneath the Dayoukeng area. Thus, the seismic conduit may be one of the major pathways for ascending magma if the volcano erupts in the future. Certainly, possibility of developing different magma pathways at other clustered seismicity zones such as beneath Mt. Chihsin might not be totally excluded because there is no particular seismicity pattern for defining magma pathways in the future eruption.

## Method

We employed double-difference seismic tomography^17^ to produce a more accurate model of the velocity and event locations for the TVG in Taiwan. Among the 8,194 earthquakes recorded at 40 seismic stations in the TVG between 2014 and 2017, we have carefully selected 2,836 events to avoid spacing redundancy within each model grid during tomographic inversion (Figs. S6 and S7). The criteria for selected earthquakes includes (1) the rms (root-mean-square) values of travel-time residuals were less than 1 sec, and (2) both horizontal and vertical errors were smaller than 2 km. Besides, in each subarea (Fig. 1), we only used one earthquake that was located with the minimum residual and distance error for the tomographic inversion later. In total, we used the absolute arrivals of 39,282 P-waves and 31,697 S-waves picked routinely by the Taiwan Volcano Observatory in Taiwan (TVO), and the relative arrivals of 149,009 P-waves and 107,407 S-waves. In the process of the tomographic inversion, different parameters were tried according to the values suggested by the double-difference seismic tomography^17^. The final results are given by the parameters below. The iteration number was 32, and the damping for both inversion of velocity structures and earthquake relocation was 75. The weighting ratio between absolute and different arrival times were reduced from 100 to 0.01. The huge number of relative arrivals of both P- and S-waves, which are about 4 times more than the absolute arrivals, significantly reduce systemic errors, thereby improving the velocity model of the TVG. Based on the inverted 3D velocity model, we successfully relocated 8,194 local earthquakes.

We slightly modified the velocity model (Table 1), which has been used for routinely locating earthquakes and further investigations^8–10,37^, as the initial 1D model for the double-difference tomographic inversion. The velocity model was divided into 11 layers with a depth interval of 0.5 km from the surface to the depth of 5 km. The nodes at the X and Y directions of the velocity models are shown Fig. 1. The grid spacing in each horizontal layer is 0.5 km covering an area of 10 km × 10 km in the TVG.

**Table 1:** One-dimensional velocity model for the Tatun volcanic area.

Depth (km)	P-wave (km/sec)	S-wave (km/sec)
Surface ~ 1.0	3.79	2.13
1.0~2.0	4.07	2.29
2.0~3.0	4.55	2.56
3.0~5.0	5.12	2.88
5.0~7.0	5.39	3.03
7.0~9.0	5.98	3.36
9.0~17.0	6.10	3.43
17.0~36.0	6.70	3.76
Below 36.0	7.80	4.38

In order to assess the effectiveness of the inversion, a checkerboard velocity model was applied to create a simulated dataset. Velocity perturbations of ±10% were applied to the checkerboard model with a grid spacing of 1.5 km in each layer. The simulated arrivals of both the P- and S-waves constructed from the actual seismicity and seismic stations in the TVG were used for the double-difference tomographic inversion. The results of both the P- and S-wave velocity structures were successfully recovered for major portions of the study area (Figs. 3 and S2). The reliable inverted result areas gradually shrank with depth due to the limitations of deeper earthquakes.

## Supplementary information

Supplementary information.

Supplementary information2.
