# Supplementary material for: Active Volcanism Revealed from a Seismicity Conduit in the Long-resting Tatun Volcano Group of Northern Taiwan
Source: Sci Rep. 2020 Apr 9;10:6153. doi: 10.1038/s41598-020-63270-7 (PMC7145833; doi:10.1038/s41598-020-63270-7)
Supplement: Supplementary file 2 — Supplementary information2. [file 41598_2020_63270_MOESM2_ESM.docx]

**Active Volcanism Revealed from a Seismicity Conduit in the Long-resting Tatun Volcano Group of Northern Taiwan**

**Pu, H.C.^1^, C.H. Lin^2,3,4,9,^*, Y.C. Lai^3,4^, M.H. Shih^2,4^, L.C. Chang^3,4^, H.F. Lee^3,4^, P.T. Lee^5^, G.T. Hong^5^, Y.H. Li^6^, W.Y. Chang^7,8^ and C.H. Lo^3,9^**

1. Seismological Center, Central Weather Bureau, Taipei, Taiwan
2. Institute of Earth Sciences, Academia Sinica, Taipei, Taiwan
3. National Center for Research on Earthquake Engineering, National Applied Research laboratories, Taipei, Taiwan
4. Taiwan Volcano Observatory at Tatun, Taipei, Taiwan
5. Central Geological Survey, Ministry of Economic Affairs,, Taipei, Taiwan
6. Industrial Technology Research Institute, Hsinchu, Taiwan
7. College of Environmental Studies, National Dong Hwa University, Hualien, Taiwan
8. Center for Interdisciplinary Research on Ecology and Sustainability, National Dong Hwa University, Hualien, Taiwan
9. Department of Geosciences, National Taiwan University, Taipei, Taiwan

(*) Corresponding author: Cheng-Horng Lin

P O Box 1-55, Nankang, Taipei, Taiwan

Email: [lin@earth.sinica.edu.tw](mailto:lin@earth.sinica.edu.tw)

Tel: 886-2-27839910 ext. 1521

Fax: 886-2-27839871

Submitted to ***Scientific Reports*** on June 6, 2019

1^st^ revised on Oct. 23, 2019

2^nd^ revised on Nov. 30, 2019

3^rd^ revised on Feb. 16, 2020

**Supplement materials:**


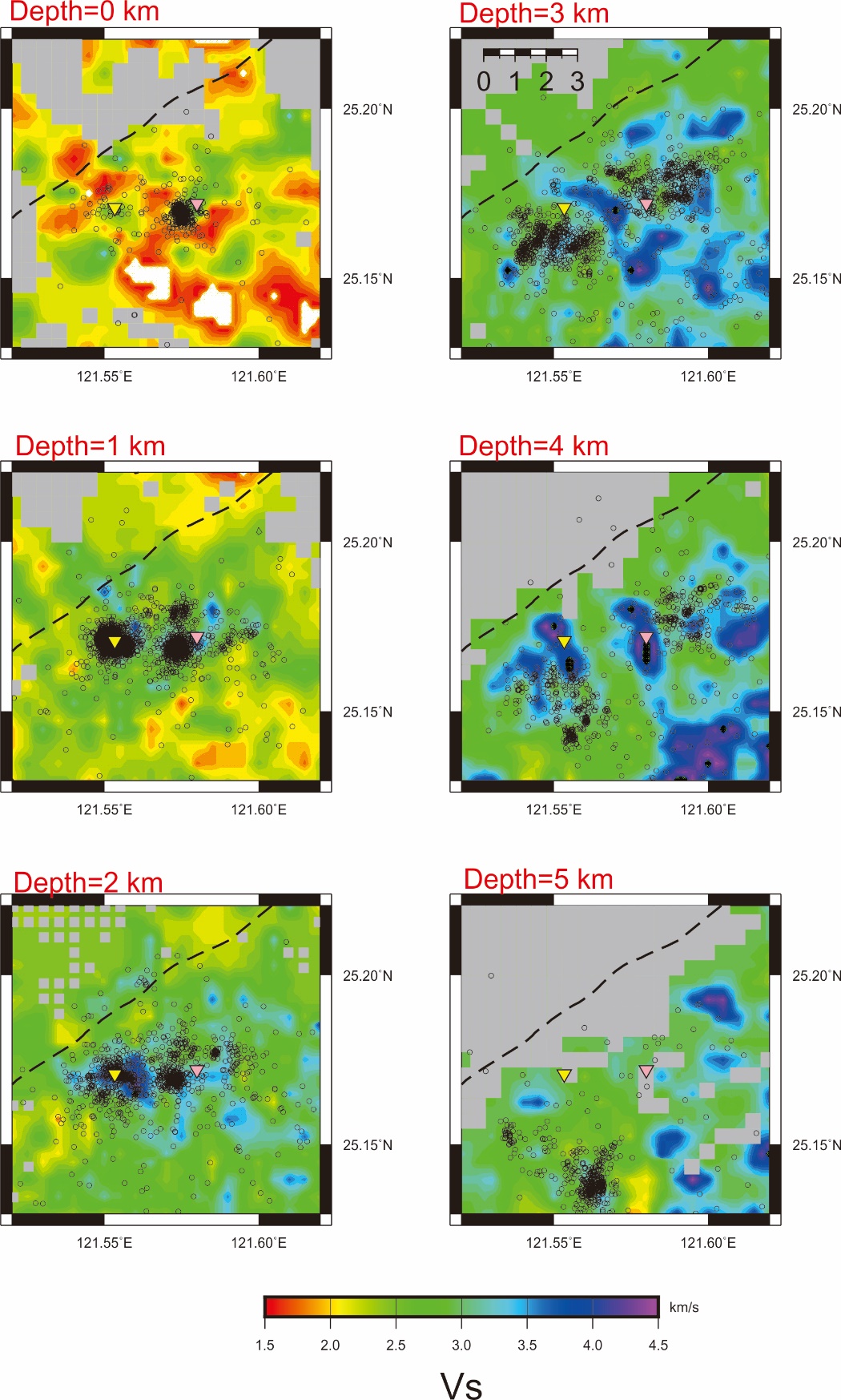


Fig. S1 S-wave Velocity structures at six layers from 0 km to 5 km. The relocated seismicity (small circles) is also plotted.


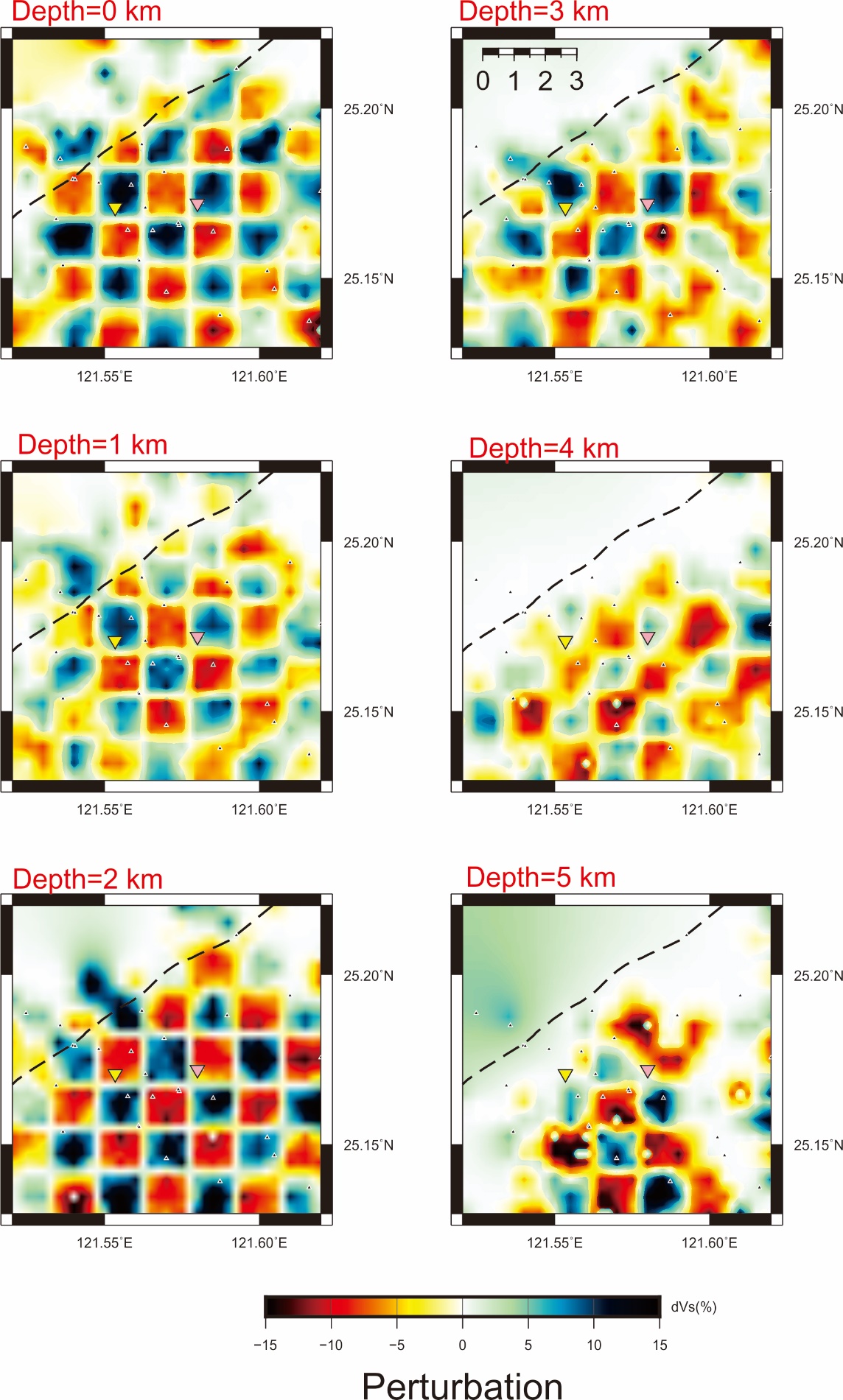


Fig. S2 The S-wave perturbations in 6 layers inverted from the checkerboard velocity model.


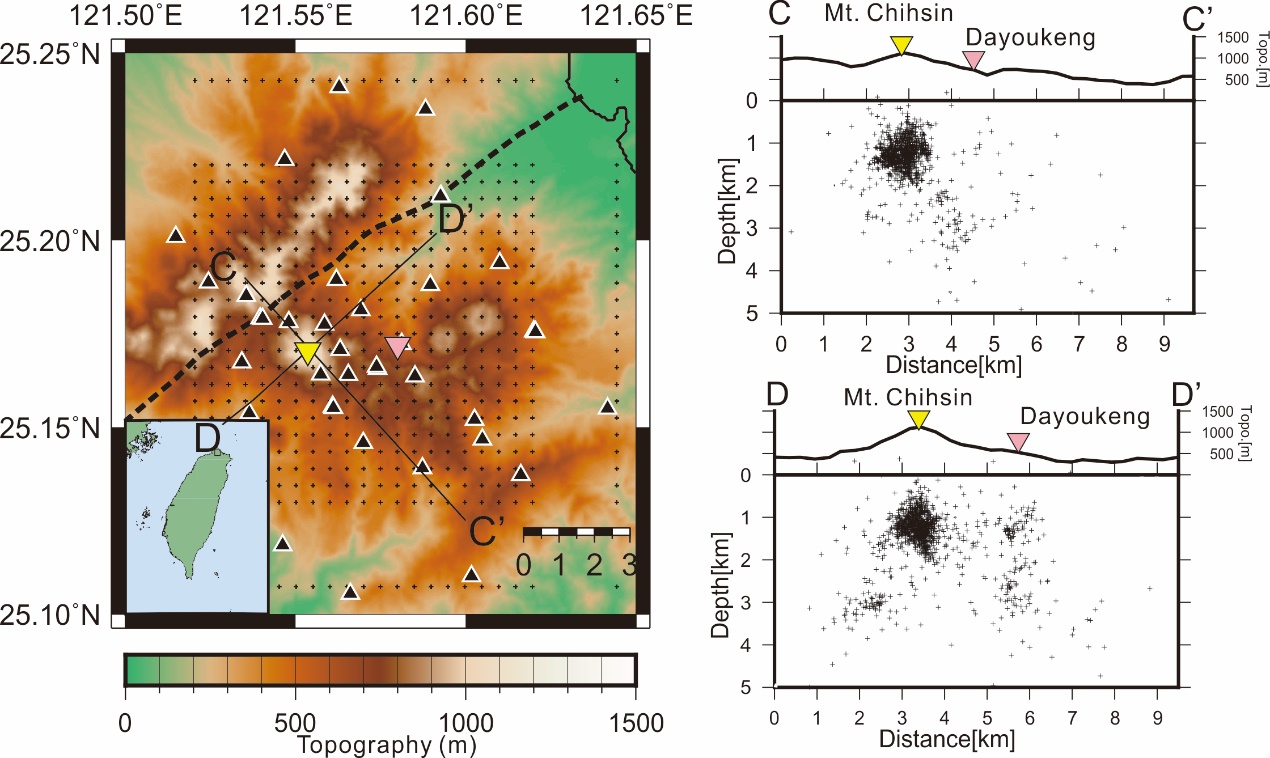


Fig. S3 Locations of the Tatun volcano group in the northern tip of Taiwan and two perpendicular depth-profiles across Mt. Chihsin. The black, yellow, and pink triangles in the left panel mark the locations of seismic stations, Mt. Chihsin, and Dayoukeng fumarole in the TVG, respectively.


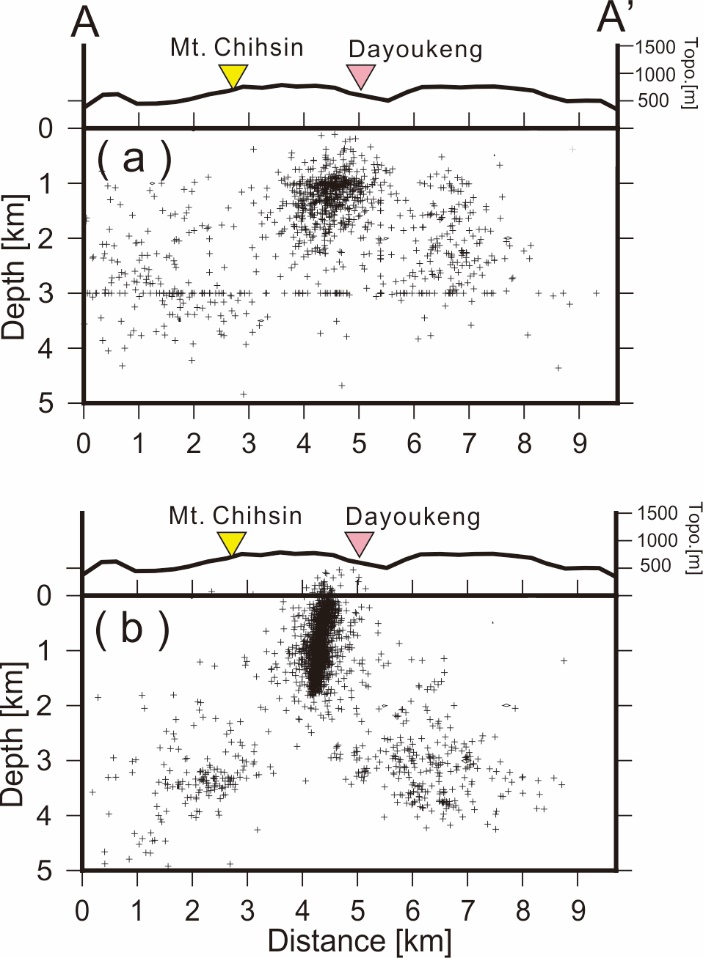


Fig. S4 Comparison of seismicity beneath the Dayoukeng area (a) before and (b) after the 3D relocation across the A-A’ profile. The relocated earthquakes with a distance of less than 0.5 km are selected to project on the profile.


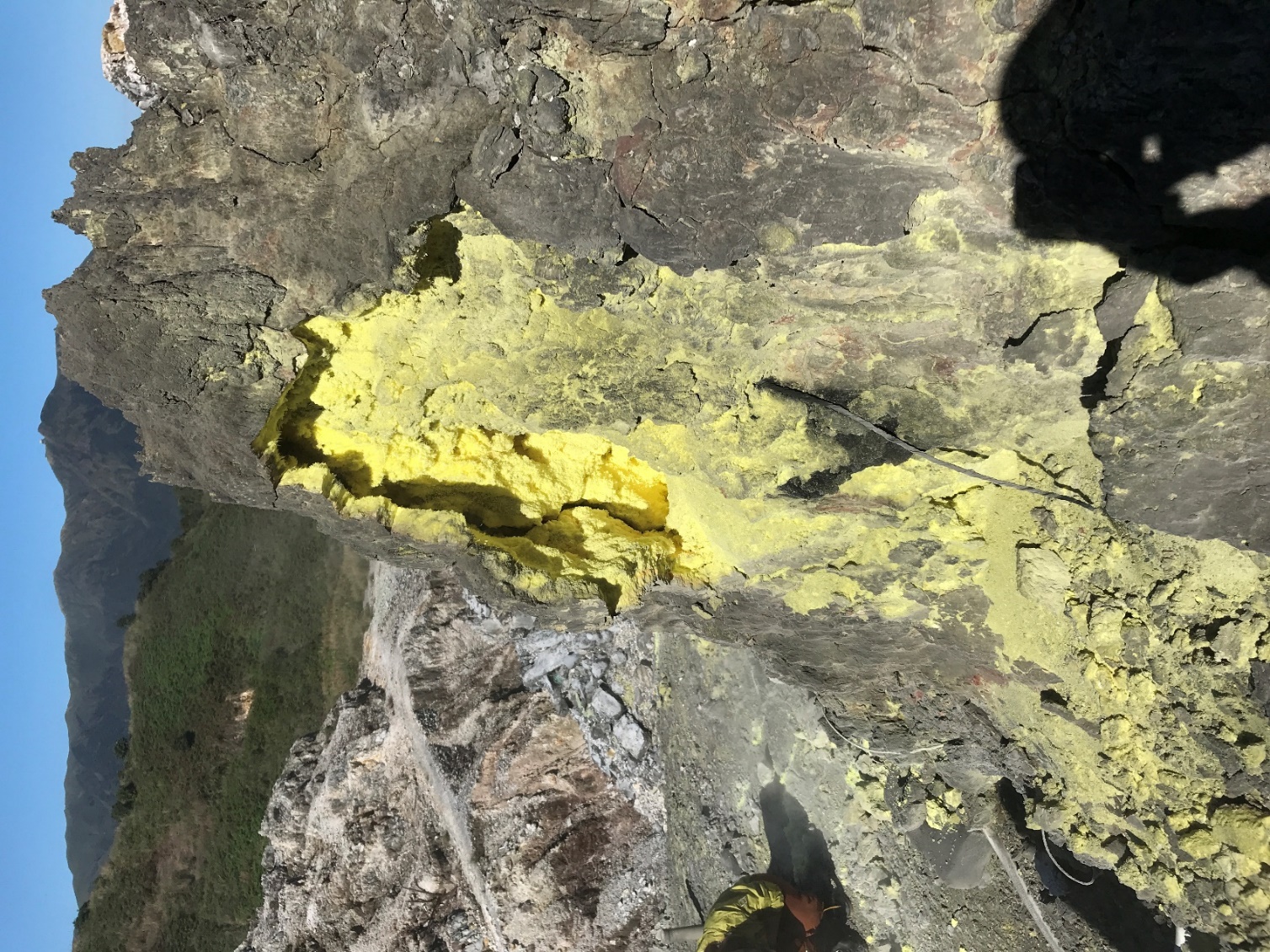


Fig. S5 One of the large sulfur-towers at the Dayoukeng fumarole in the TVG of northern Taiwan.


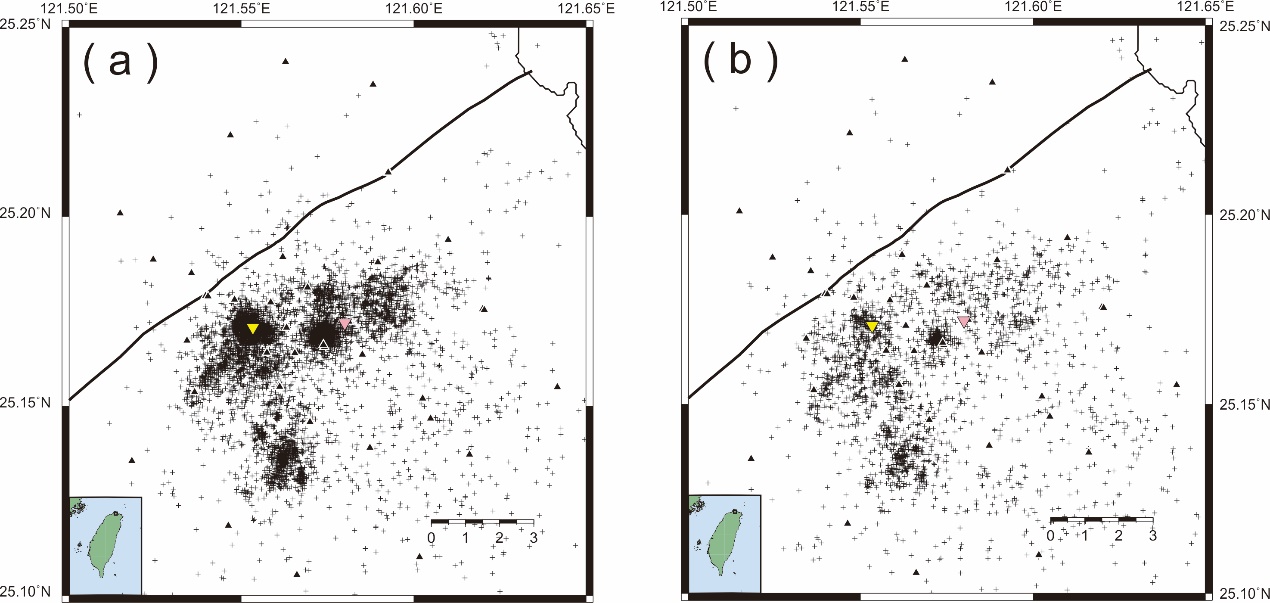


Fig. S6 Seismicity of (a) 8,194 earthquakes detected in 2014-2017 and (b) 2,836 selected earthquakes for doing the tomographic inversion.


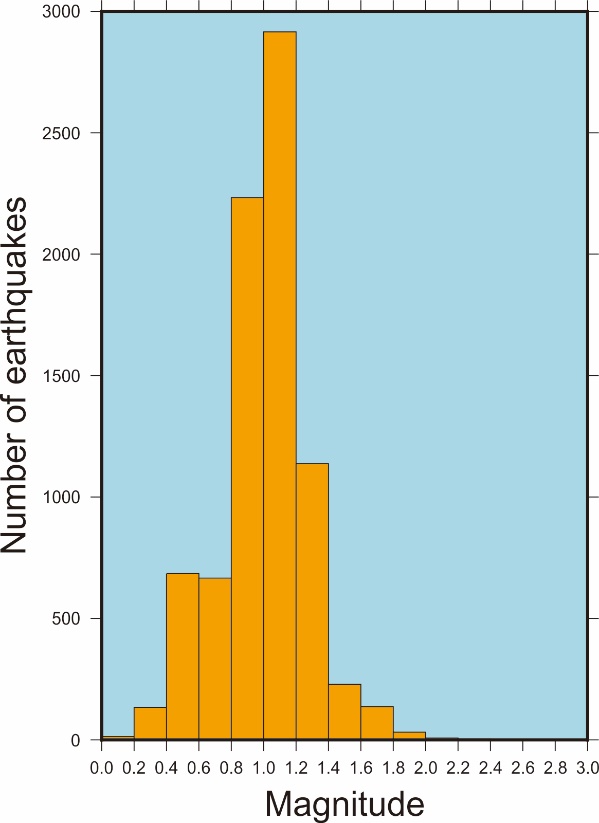


Fig. S7 Statistic plot of earthquake magnitude distribution in the TVG in the period between 2014 and 2017.

Supplement Movie: Strong degassing at the Dayoukeng fumarole in the TVG.
